# Adapting Dementia Care Management to a Regional German Context: Assessment of Changes in Acceptability, Appropriateness, and Feasibility

**DOI:** 10.1177/07334648241258024

**Published:** 2024-06-05

**Authors:** Katja Seidel, Lena Rupp, Jochen René Thyrian, Julia Haberstroh

**Affiliations:** 1 Department of Psychology, Psychological Aging Research, Faculty V: School of Life Sciences, 14312 University of Siegen, Siegen, Germany; 2 581365 German Center for Neurodegenerative Diseases (DZNE), Site Rostock/Greifswald, Greifswald, Germany; 3 Institute for Community Medicine, 163285 University Medicin Greifswald, Greifswald, Germany

**Keywords:** dementia care, care management, participatory research, implementation research

## Abstract

Dementia care management, an evidence-based care concept in Germany, optimizes care for people with dementia and their caregivers. Implemented by qualified professionals, it comprises intervention modules addressing treatment and care, medication management, and caregiver support. Positively evaluated in one federal state, it’s recommended for nationwide integration into routine care. Since the infrastructure of the German healthcare system differs regionally, the concept underwent adaption for regional implementation in a participatory, iterative process. Five local healthcare experts as co-researchers tested and adjusted selected components of the concept in a pilot study. This trend analysis aims to assess the adapted concept for acceptance, appropriateness, and feasibility. A total of 89 intervention modules were tested over 18 weeks, and the co-researcher’s assessment was gathered through an accompanying online survey. The participatory process itself was rated positively overall, but technical problems had a negative impact on the implementation and evaluation of the care concept.

**What this paper adds**

• This study stands out as one of the very few worldwide that consistently engages stakeholders, individuals with dementia, and their family members right from the outset in the implementation process, and meticulously assesses these efforts.
• Despite being a pilot study, this pioneering approach offers preliminary insights into the feasibility and advantages of participatory research for achieving successful implementation.
• The paper demonstrates the importance of a process-accompanying evaluation in order not to jeopardize the effectiveness of the actual intervention and their positive effects on the health and quality of life of older people due to implementation-related barriers.

**Application of study findings**

• Future dementia research paradigms should routinely incorporate stakeholders to promote successful implementation.
• The study findings contribute to the sustainable implementation of Dementia Care Management in nationwide healthcare in accordance with the German national dementia strategy.

## Introduction

Dementia care management (DCM) is an evidence-based collaborative model of care that aims to provide individualized and effective care for people with dementia (PwD) and caregivers within the existing established healthcare system in Germany through intersectoral, multimodal, and multiprofessional strategies (Eichler, Thyrian, Dreier et al., 2014; Eichler, Thyrian, Fredrich et al., 2014; Michalowsky et al., 2019; Thyrian et al., 2016a; Thyrian et al., 2017b). DCM has been adapted for various settings (Klein et al., 2021; Nikelski et al., 2019). The *DelpHi-standard* of optimum care within DCM was developed in accordance with German guidelines for evidence-based diagnoses and treatment of dementia, a literature review, expert meetings in the field, and a scientific advisory board (Eichler, Thyrian, Dreier et al., 2014). DCM consists of three major *pillars*: management of treatment and care, medication management, and caregiver support and education (Eichler, Thyrian, Dreier et al., 2014). Every *pillar* comprises eight different *action fields* which include a total of 25 *foci*, which consist of specific DCM *intervention modules* (Seidel et al., 2022). An example for an intervention module is an in-depth assessment of depression (Mini International Neuropsychiatric Interview, Lecrubier et al., 1997) as part of the *focus* psychiatric care within the *action field* medical diagnosis and treatment. That *action field* belongs to the *pillar* management of treatment and care.

The centerpiece of DCM is the implementation by specially qualified professionals, the dementia care managers (DeCMs). While qualification of the DeCMs is one key component, another one is the use of a specifically developed computer-software, the so-called Intervention Management System (IMS). It uses pre-defined algorithms that recommend specific *intervention modules* based on the needs provided by the PwD and caregiver. The needs assessment comprises socio-economic, medical, nursing, psychosocial, and caregiving related data (for more details, see (Eichler, Thyrian, Fredrich et al., 2014). In a care situation, DCM works as follows: Based on the comprehensive and computer-supported assessment, the DeCMs develop an individualized care plan in cooperation with the PwD, carers, and the GP (Eichler, Thyrian, Dreier et al., 2014) as the main responsible person for outpatient treatment in Germany (Purwins et al., 2023) and support and monitor the implementation of this care plan. Due to this individual and needs-oriented approach, there are no requirements regarding the frequency of visits, the duration of a counseling session, or the duration of the support provided by the DeCMs. The *DelpHi-standard* was evaluated in a cluster-randomized controlled intervention trial in a federal state in Northeastern Germany as an accepted, efficient, and cost-effective optimized care model (Michalowsky et al., 2019; Thyrian et al., 2016b, 2017a). This scientific evidence has influenced the German National Dementia Strategy to recommend DCM for nationwide implementation into routine care (Federal Ministry for Family Affairs, Senior Citizens, Woman and Youth & Federal Ministry of Health, 2020). This has not yet been realized.

Implementing such a complex multilevel intervention presents unique challenges when implemented in specific local context. Among others, German regions differ significantly in their healthcare infrastructure; there is a difference in access, availability, and utilization between urban and rural areas. One approach to meeting these challenges is the involvement of local stakeholders’ expertise. This expertise includes target-group-specific insights into regional and context-specific factors. Various authors assume that involving stakeholders through participatory methods can improve the likelihood of successful implementation of needs-based interventions (Boekholt et al., 2020; Ramanadhan et al., 2018; Schlechter et al., 2021; Wright & Kongats, 2018). Involving stakeholders has improved studies’ acceptability, feasibility, rigor, and relevance (Forsythe et al., 2019; Jiménez-Chávez et al., 2018; Maurer et al., 2022). Maurer and colleagues found stakeholders’ impact to be dynamic, non-linear, and iterative throughout the study process (2022). Furthermore, stakeholders themselves indicate and advocate a participatory approach allowing a high degree of networking prior to and facilitating successful implementation of evidence-based concepts (Seidel et al., 2022). Bethell and colleagues recommend more research specifically about the impact of participatory approaches in dementia settings (Bethell et al., 2018). There is a need to gain more and deeper insights of the impact of participatory methods on the implementation of complex health interventions in dementia care.

**Figure 1.**
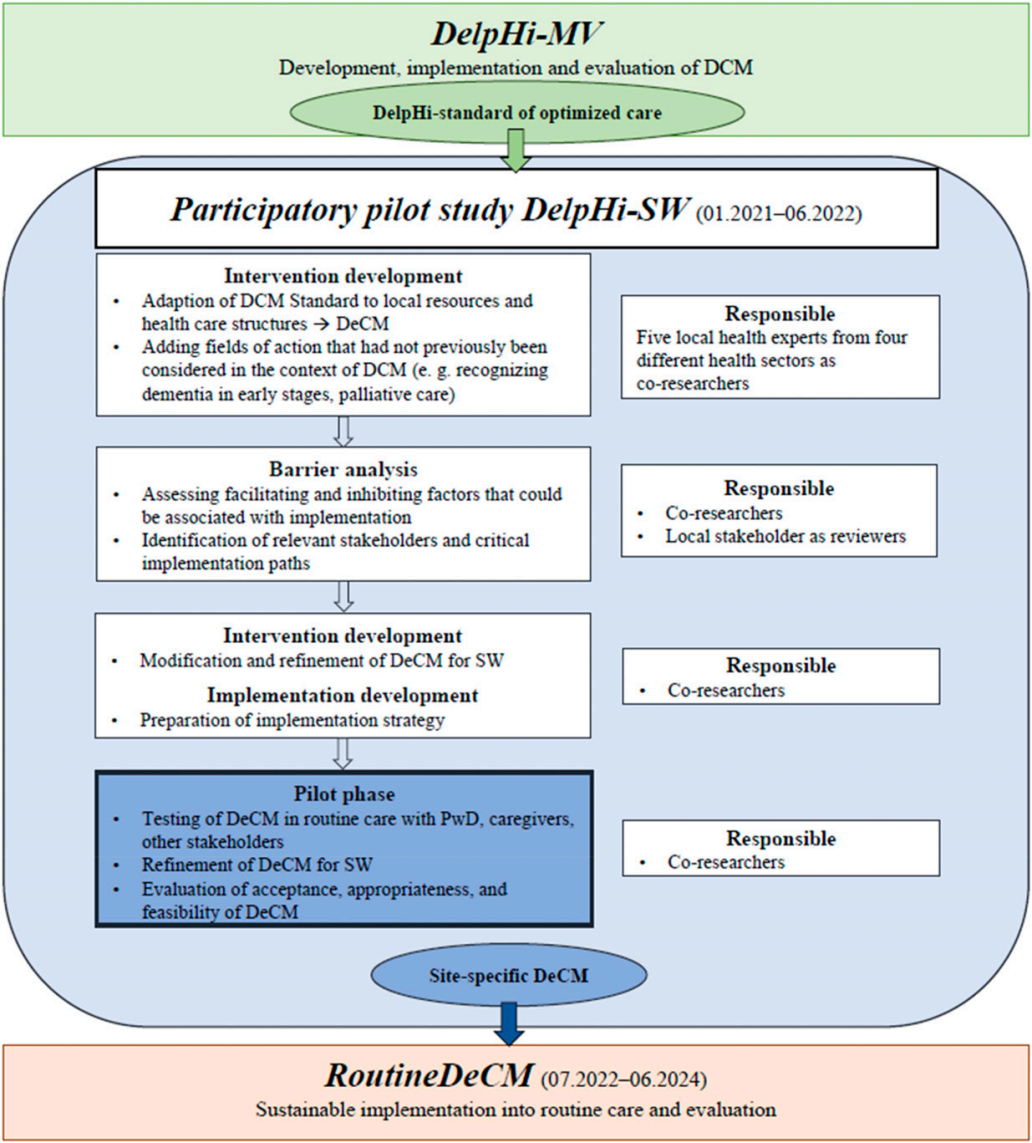
Embedding of the pilot phase within the participatory pilot study DelpHi-SW. *Note*. DelpHi-MW, Dementia: life- and person-centered help in Mecklenburg-West Pomerania; DelpHi-SW, Dementia: life- and person-centered help in Siegen-Wittgenstein; DCM, original dementia care management model; DeCM, adapted version of dementia care management model; PwD, people with dementia; SW, Siegen-Wittgenstein. In all phases, the academic researchers assumed the role of facilitators. Adapted and updated to Aadapting a Dementia Care Management Intervention for Regional Implementation: A Theory-based Participatory Barrier Analysis, by Seidel et al. (2022), *International Journal of Environmental Research and Public Health*, *19*(9), 5478, p. 2. (https://doi.org/10.3390/ijerph19095478).

The study presented here is part of the participatory pilot study DelpHi-SW (Dementia: life- and person-centered help in Siegen-Wittgenstein). It aims to adapt and implement DCM into the routine care of an exemplary local setting (Siegen-Wittgenstein: SW), using a structured participatory approach featuring four steps (Purwins et al., 2023; Seidel et al., 2022). Figure 1 (adapted and updated to Seidel et al., 2022, p. 2) depicts the participatory procedure and where the present study (pilot phase) is embedded within DelpHi-SW. In the adapted version, the original DCM is abbreviated as DeCM (Seidel et al., 2022).

The pilot study as the final step is therefore an essential implementation activity. A successful implementation process is a necessary and important but not sufficient prerequisite for the success of the actual intervention (Proctor, 2020). Implementation outcomes are key measures and indicators that can be used to describe the mechanisms and success of implementation efforts (Proctor, 2020). Therefore, a key focus of the pilot phase was the question of how the participatory adaption process relates to the evaluation of the acceptability, appropriateness, and feasibility as established implementation outcomes of the adapted model by the co-researcher as relevant stakeholders. In this article, we present the results of this question.

## Methods

### Design

The adapted model was piloted by the DeCMs in routine care between February and June 2022 (18 weeks). The accompanying analysis of acceptability, appropriateness, and feasibility follows a participatory longitudinal design using quantitative data from an online survey.

### Participatory Co-Researchers

The research team consisted of academic researchers and five co-researchers. Co-researchers (4 females and 1 male) were specifically qualified DeCMs based on the available curriculum (Dreier et al., 2011). In addition, they were stakeholders and local healthcare experts from four different healthcare sectors (SK, Alzheimer’s society, MK, local medical network, HB, care and welfare association, and MB and LB, hospital). Their responsibility comprises the co-decision in the planning, data collection, evaluation of the implementation outcomes, interpretation of results, and reviewing the article. The academic researchers built an interdisciplinary team of psychologists (KS and LR) a gerontologist, and one IT-expert. Additionally, two advisory boards, consisting of PwD and caregivers, were available to provide advice throughout the entire piloting period. However, they were not involved in the evaluation presented in this article.

### Participatory Procedure

**Figure 2.**
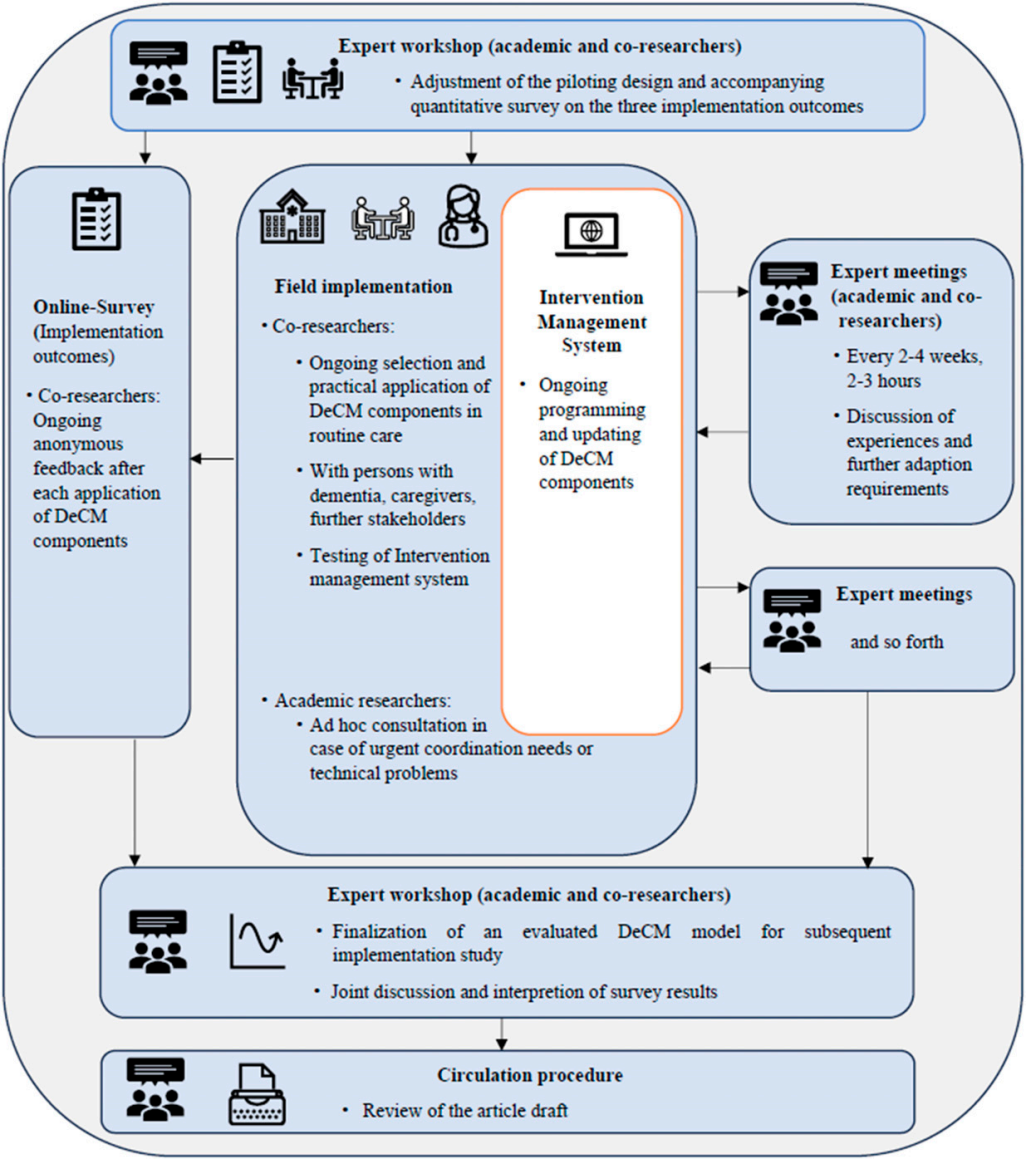
Participatory, iterative procedure during the piloting phase. *Note.* Field implementation = 18 weeks. DeCM, dementia care management.

The piloting was carried out in an iterative, participatory process (Figure 2). After an expert workshop, the co-researchers tested the practical implementation of selected DeCM components in routine care. A total of 22 people (PwD, caregivers, and additional experts from their work context) were recruited in the context of co-researchers’ clinical work and involved in the piloting. An ethical review and approval were obtained from the Council of Research Ethics of the University of Siegen. To ensure informed consent of PwD, the study material was previously adapted by the PwD advisory board in terms of dementia-sensitive language. PwD and caregivers were either counseled at home or in counseling facilities. Other experts as additional stakeholders were introduced to selected DeCM components.

Regular expert meetings were conducted during the pilot phase. Additionally, co-researchers provided anonymous feedback through an online survey (see *Data Collection and Instruments*). Simultaneously, the adapted DeCM intervention modules were programmed and integrated into the IMS throughout the entire pilot period (see Table 1 for a comparison between the original DCM and the adapted version, DeCM). Toward the conclusion of the pilot phase, the outcomes within the scope of the current study were compiled by the academic researchers. These results were then jointly interpreted and discussed with the co-researchers, who acted as the ultimate decision-makers and held responsibility for the adapted DeCM version at the conclusion of the piloting phase. Aligned with the stages of participation, this level of involvement reflects genuine participation (Bethmann et al., 2019). In all phases, the academic researchers assumed the role of facilitators. Throughout the piloting phase, their responsibilities included preparing and overseeing expert meetings, establishing communication channels, and taking charge of technical implementations and adjustments.

**Table 1.** Exemplary Comparison of the Original Delphi-Standard of Optimum Care (DCM) and the Adapted Version (DeCM): Intervention Modules and Specific Interventions Automatically Recommended by the Intervention Management System When Relevant Trigger Conditions are Met.

	Focus	Intervention Module	Trigger Condition	Specific Intervention
DelpHi-standard of optimum care	Indication check anti-dementia medication	Evaluation of the application of anti-dementia medication	If no anti-dementia medication is yet prescribed and there is a request	Initial information and referral to the general practitioner/neurologist
	Prevention of drug related problems	Execution of medication management	If a medication check was requested within the framework of the medication anamnesis	Forwarding medication documentation
	Help with application of medicine	Recommendations for medication storage	If medication is found open AND/OR stored in bathroom within the framework of the medication anamnesis	Recommendations for the storage of medication, including the proper storage of medication that requires refrigeration
		Recommendation for medication dispenser	If DeCMs recommends medication dispensers	Recommendations for the purchase and utilization of a medication dispenser
Adapted version	Indication check antidementives	Evaluation of the application of anti-dementia medication	If no anti-dementia medication is yet prescribed AND/OR there is a request	Initial information and referral to the general practitioner/neurologist
	Medication check	Prevention of drug related problems	If behavioral problems occur after medication intake	Observation advice
			If DeCMs suspect side effects AND/OR interactions	Arrange for an external review of the current medication list and application form
			If medication can no longer be taken independently	Engage a care service
			If current form of application is not possible	
		Recommendations for medication storage	If medication is found open AND/OR stored in the bathroom within the framework of the medication anamnesis	Recommendations for the storage of medication, including the proper storage of medication that requires refrigeration
			If DeCMs recommend medication dispensers	Checking and documenting the expiry date
		Comparison of medication plan with existing medication	If the medication plan and existing medication do not match	Informing general practitioner and request an actualization
				Dispose of old medications
		Help with application of medicine	If medication can no longer be prepared independently AND/OR the patient wishes to prepare it themselves, but there is an objective need for assistance	Consultancy: Professional implementation by outpatient care service
			If DeCMs recommends medication dispensers	Advice on smart memory aids and recommending purchase and utilization of a medication dispenser

*Note.* The exemplary comparison refers to the *pillar* Medication Management, *action field* Pharmaceutical Treatment and Care. DeCMs, dementia care managers.

### Data Collection and Instruments

Three established implementation outcomes according to the Taxonomy of Outcomes for Implementation Research by Proctor et al. (2011) were used to assess the success of the participatory approach during pilot phase as an early implementation stage: acceptability, appropriateness, and feasibility. Acceptability refers to the perception of an innovation as satisfactory by the implementation stakeholder; appropriateness describes the perceived fit of an innovation within a practical setting and given issue; and feasibility describes the extent to which an innovation can be successfully realized within a certain setting (Proctor et al., 2011; Weiner et al., 2017). The German version of three implementation outcome measures was adapted for the use in this analysis (Kien et al., 2021; Weiner et al., 2017). Neither norms nor thresholds for interpretation are available, but higher values indicate greater acceptance, appropriateness, and feasibility (Weiner et al., 2017).

Co-researchers were briefed on the conceptual underpinnings of the implementation outcomes. In response to their feedback indicating that the time needed to respond to the entire scales would be excessively long, the scale underwent further adaptation. To enhance the probability of completion and adherence, a decision was made to present only one item per scale, guided by psychometric criteria. The item with the highest discriminatory power or factor loading was then chosen (Kien et al., 2021; Weiner et al., 2017). The wording of the items was adapted to the specific implementation context: “The questionnaire/assessment or the DeCM intervention module meets my approval” (acceptability), “The questionnaire/assessment or the DeCM intervention module seems fitting” (appropriateness), and “The questionnaire/assessment or the DeCM intervention module seems doable” (feasibility). The assessment was conducted using the online survey tool LimeSurvey (“LimeSurvey”) which was presented during a kick-off meeting and adapted according to the co-researchers’ change requests.

The co-researchers completed the online questionnaire after each application of DeCM components in routine care. They were asked to indicate their agreement to each item on a 5-point Likert scale ranging from “completely disagree” (1) to “completely agree” (5). In case their response was “neither, nor,” “rather disagree” as well as “completely disagree,” there was an additional free text field to allow their further explanations and in-depth feedback. On average, the results in LimeSurvey were checked twice a week by the research team, the comments and topics in the free text fields were made available as a preparation for the expert meetings and with the aim of enriching and explaining the results of the quantitative survey. In addition, regular reminders were provided regarding the online survey.

### Data Analysis

The quantitative data was edited and analyzed by the academic researchers using SPSS (“IBM SPSS Statistics for Windows,” 2022). To gauge and assess the impact of the participatory approach on the three implementation outcomes—acceptability, appropriateness, and feasibility—a trend analysis was conducted using aggregated data from the sample. Trend analysis, employed as a process-analytical method, offers a valuable framework for describing and scrutinizing the progress of variables over time within specific mathematical functions (Schall, 2012). This approach is particularly advantageous in intervention research, especially when dealing with smaller sample sizes, as demonstrated in prior studies (Kolling et al., 2013; Schall et al., 2015). By utilizing trend analysis, we aim to capture nuanced changes and patterns over the course of the study, providing a comprehensive understanding of the evolving dynamics related to the implementation outcomes. This methodological choice aligns with the recognized effectiveness of trend analysis in uncovering subtle trends in data, thereby enhancing the robustness of our evaluation.

We computed the weekly means from all online ratings for each of the three outcome variables with the break variable time. This results in a sequence of weekly mean values that represent the mean course of the sample over time. That means we obtained the mean course of acceptability, appropriateness, and feasibility across all ratings and over the survey period. No weekly mean values could be determined for six single weeks, as no testing in the field and consequently no evaluations were conducted due to missing participant appointments and/or holidays. Presuming that the missing values cannot be explained by other variables during the study (missing completely at random, MCAR) (Kwak & Kim, 2017) they were replaced using linear interpolation as estimation method to replace missing values. The trend analyses for acceptability, appropriateness, and feasibility should help to clarify whether the time series follows a significant trend.

## Results

Over 18 weeks of field implementation, 89 DeCM components (11 DeCM intervention modules and 78 assessments and tests) were trialed in 12 weeks. In these, between 1 and 17 online assessments per week were made (*M* = 7.42; *SD* = 5.35). For 6 weeks, no data points were collected. Based on the aggregated data, the weekly means and standard deviations are as follows (linear interprojected weekly mean values in brackets): acceptability, *M* = 3.12; *SD* = 0.86 (M = 3.04; *SD* = 0.82); appropriateness, *M* = 3.03; *SD* = 0.74 (*M* = 2.91; *SD* = 0.70); and feasibility, *M* = 2.59; *SD* = 0.85 (*M* = 2.51; *SD* = 0.74).

We examined change in acceptability, appropriateness, and feasibility over time (weeks) using regression models with polynomial time trends of varying degrees. We compared linear, quadratic, cubic models, and a model with a polynomial of degree four against each other, respectively. Model comparisons were done (a) formally by means of the residual sum of squares reduction test, and (b) descriptively by change in R-squared.

**Figure 3.**
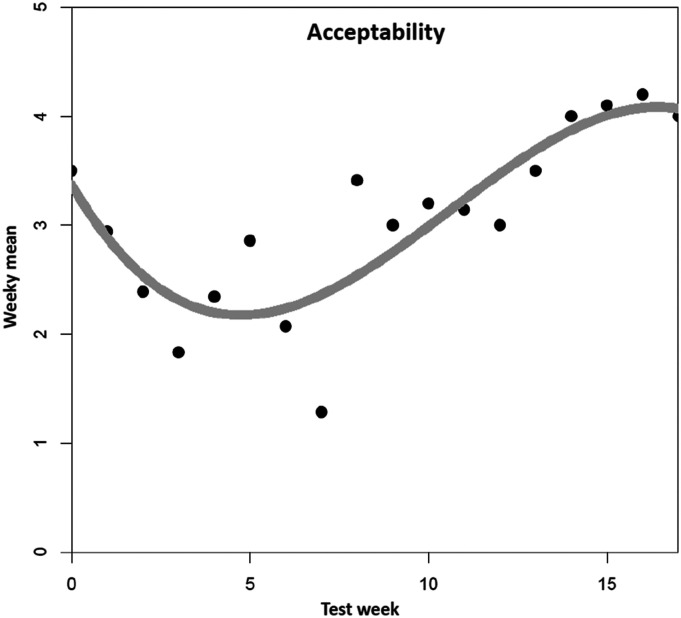
Mean trend for acceptability. *Note. N* = 89 tested dementia care management components (*n* = 11 DeCM intervention modules, *n* = 78 assessments and tests). A total of 22 people (PwD, caregivers, and additional stakeholders) were involved in the field implementation. The data was collected using a 5-point Likert scale ranging from “completely disagree” (1) to “completely agree” (5). No weekly mean values are available for six individual weeks (2, 3, 5, 7, 14, and 16). The missing values were replaced by linear interpolation as an estimation method.

For acceptability (Figure 3), the cubic model had the best fit to the data and showed a significant improvement in variance explained (*R*^2^ = .72) over both the linear model (*R*^2^ = .42) and the quadratic model (*R*^2^ = .62), respectively (all *p <* .05). Additionally including higher degrees did not further improve model fit (*F*(1, 13) = 0.23, *p = .*64). The coefficients of the cubic model are as follows: intercept (3.37, *p* < .001), linear time trend (−0.56, *p* = .01), quadratic time trend (0.08, *p* = .01), and cubic time trend (−0.002, *p* = .04), denoting an average decrease of acceptability at the very beginning, followed by an increase, and finally an almost constant phase at the end of the trajectory.

**Figure 4.**
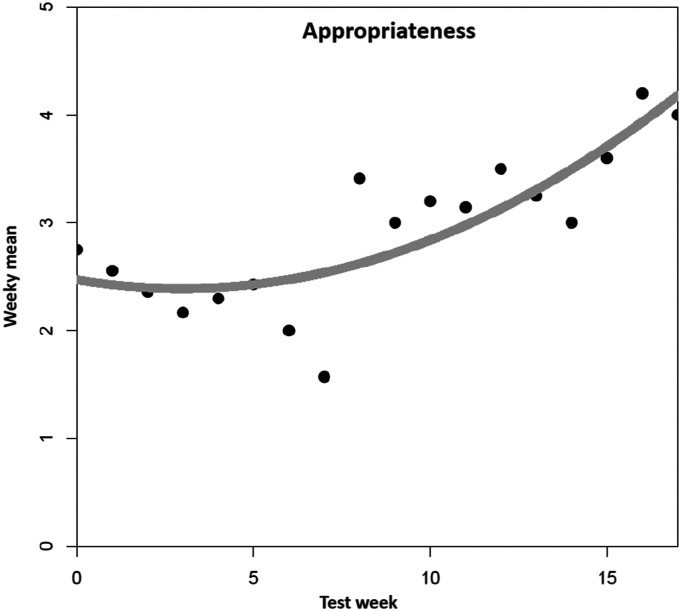
Mean trend for appropriateness. *Note. N* = 89 tested dementia care management components (*n* = 11 DeCM intervention modules, *n* = 78 assessments and tests). A total of 22 people (PwD, caregivers, and additional stakeholders) were involved in the field implementation. The data was collected using a 5-point Likert scale ranging from “completely disagree” (1) to “completely agree” (5). No weekly mean values are available for six individual weeks (2, 3, 5, 7, 14, and 16). The missing values were replaced by linear interpolation as an estimation method.

For appropriateness (Figure 4), the quadratic model had the best fit among the examined models, and significantly enhanced fit in comparison to the linear model (*F*(1,15) = 5.05, *p =* .04). R-squared increased from .58 (linear model) to .68 (quadratic model). Including a cubic trend would have increased R-squared only by another insignificant 3.7% (*F*(1,14) = 1.83, *p* = .20). The estimate for the intercept was 2.47 (*p* < .001), for the linear time trend −0.05 (*p* = .47) and for the quadratic term 0.01, (*p* = .045), denoting a negligible decrease in appropriateness at the beginning followed by a monotone increase throughout the remainder of the observed timespan.

**Figure 5.**
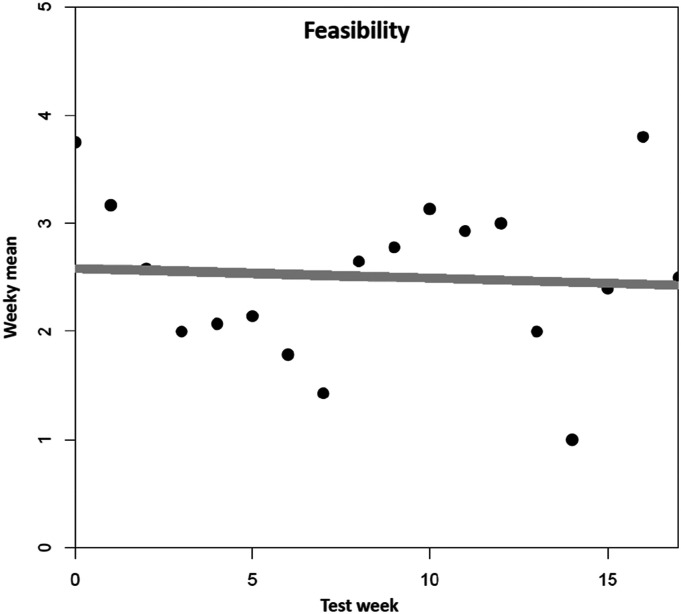
Mean trend for feasibility. *Note. N* = 89 tested dementia care management components (*n* = 11 DeCM intervention modules, *n* = 78 assessments and tests). A total of 22 people (PwD, caregivers, and additional stakeholders) were involved in the field implementation. The data was collected using a 5-point Likert scale ranging from “completely disagree” (1) to “completely agree” (5). No weekly mean values are available for six individual weeks (2, 3, 5, 7, 14, and 16). The missing values were replaced by linear interpolation as an estimation method.

For feasibility (Figure 5), neither of the polynomial models improved fit over the intercept-only model (all *p* > .10). There are not any clear time trends, and we observe only random fluctuation around the average value of 2.50.

## Discussion

This analysis aimed to investigate how a participatory approach to the implementation of DeCM leads to a version that is accepted by those involved and is considered appropriate and feasible. Regarding acceptability, its level was higher at the beginning, decreased by the end of the second month of field implementation (three weeks of testing) and increased again in the last month. Similar patterns were found regarding the assessment of perceived appropriateness. In the first tests, the DeCM intervention modules/assessments were perceived as rather inadequate. As with acceptability, this assessment reached its lowest point at the end of the second month of testing, only to rise again to higher level in the final weeks. At the end of the pilot period, the co-researcher felt that the finalized DeCM version was rather appropriate for the intended purpose. In terms of feasibility, the co-researchers considered it to be rather given at the beginning; however, the rates decreased by the end of the second month too. After the assessment had settled at a medium level by the end of the 13th week of piloting, DeCM was assessed as completely unfeasible in the 15th week.

In the joint discussion of these findings, co-researchers stated that the weak start of the piloting was a cause for rapid decline in acceptance after motivation and satisfaction had been very high at the beginning. According to them, the main reason for their dissatisfaction was the IMS. Due to the perceived insufficient technical conditions, the co-researchers decided to initially test only with colleagues instead of PwD and carers. Furthermore, relevant assessment instruments only had been gradually introduced into the IMS. Almost only assessments were tested because intervention modules were only introduced at the end of the pilot phase. The increase of acceptability in the last third of the pilot period can be explained by the fact that only minor errors occurred at the end due to the iterative joint adaptation process and the increased feedback loops. In addition, the frequent application may have led to more routine and confidence over time. Co-researchers stated that an appropriate skillset with corresponding degrees of freedom in decision-making lead to a high level of acceptability at the end of the piloting.

Regarding the perceived appropriateness of DeCM, the co-researchers considered various influencing factors. They stated that the DeCM qualification failed to create a basic understanding of the principle of assessment-based interventions. Additionally, the assessments which were more likely used in a scientific context would be less appropriate and relevant in a practice context. Over time, the perception of the co-researchers changed because of increased routines, more support, and the intensified feedback loops from the project team. Thus, the perception tended more and more toward “DeCM is round, it fits” which is illustrated in increased rates of appropriateness. Furthermore, the co-researchers found it difficult to establish an appropriate self-concept and subsequently become an appropriate role model as DeCMs. This would have been important to convince oneself, PwD and caregivers of the benefits and appropriateness of DeCM. This lack of an appropriate self-concept would most likely have been reflected in the perception and evaluation of acceptability and appropriateness.

In terms of feasibility, many DeCM components could not be tested at all or only unreliably with system crashes, or bugs. Furthermore, some assessments were rated too impractical in terms of time and effort. Overall, the adaptation was perceived as unfinished, which is why its feasibility was not considered to be given.

Overall, co-researchers drew a positive conclusion regarding the participatory approach *itself* which was at least positively reflected in the assessment of acceptance and appropriateness of the DeCM at the end of the piloting phase. It was described as different from other study experiences where all decisions were made by research teams. Thus, the possibility to have choices and equal, significant decision-making power was a positive experience. This led to a self-perception among the DeCMs that they, as practice partners had “the freedom to make their own decisions as needed” and “helped to design the final product.” This assessment is in line with previous research findings suggesting that participatory research enables co-learning between people affected, healthcare providers and researchers which results in in-depth clinical understanding of the disease (Jiménez-Chávez et al., 2018; Staley & Minogue, 2006) and increased researchers’ knowledge when working with PwD (Kowe et al., 2022). Stakeholder input can reduce health disparities and distribute power equally therefore benefiting people who are otherwise often marginalized in academic settings (Jiménez-Chávez et al., 2018; Staley & Minogue, 2006).

However, this general positive assessment of the influence of the participatory approach by our co-researchers can only be found to a limited extent in our data for all three implementation outcomes. Additionally, co-researchers formulated the assumption that the tendency toward a positive trend for acceptability and appropriateness would not have continued beyond June. For both, they assumed a decrease after the last DeCM components were introduced in the IMS, as the error rate would then have increased again. It is assumed that afterward, however, a positive trend in all outcomes would have occurred, since by then, almost all errors in the IMS had been eradicated and they would be now personally convinced of the final DeCM version. One could assume that the technical challenges seemed to have overshadowed stakeholder’s perceptions of other components or processes that worked well. One possible explanation could be the cognitive bias called negativity bias (Rozin & Royzman, 2001) which postulates that humans commonly give greater weight to negative entities which might have biased the results of our implementation outcomes.

### Limitations

This analysis has some limitations. Our study had an explorative character with no experimental or controlled design which implies a lack of conclusive results. Further, we cannot rule out potential response biases due to the data assessment procedure. Experts in the work context might have answered favorably (social desirability bias) or not truthfully (response bias) when interviewed by their co-researching work colleagues. When tested with persons in the home, disruptive factors related to this specific environment could have occurred (e.g., distractions that led away from the topic). The piloting period of 18 weeks was due to the timeframe of the project and is relatively brief. Additionally, measurement points were omitted during the participatory process, meaning that only a limited number of 12 measurement points are available. This reduces the variability and conclusiveness of our data. There seem to be a lack of a conceptual distinction between acceptability and appropriateness in the online survey. Furthermore, the project-related technical difficulties with the IMS seemed to be more responsible for the extent and course of the trends than the participatory approach.

### Strengths

A strength of the study lies in the comprehensive participatory approach. Empowerment, clarity and transparency, accountability, equal opportunities, and commitment to change were named as guidelines for participatory research (Staley & Minogue, 2006). All those principles were reflected in the set-up of research project. The stakeholders were empowered to participate in various steps of the research process. Also, all feedback rounds and meetings occurred regularly providing room for clearing up questions or challenges thus providing clarity and transparency. When feedback was given, the academic and technical team showed efforts to find solutions which stands for accountability. Every stakeholder was given equal opportunities to participate and test the components of DeCM. Also, feedback was taken immediately into account and processes were adapted which signifies commitment to change. In the joint interpretation process, the different perspectives, and perceptions of the participating academic and co-researcher became visible and understandable. This can lead to a better understanding of the phenomenon under study, as such a shared sense-making “can uncover the richness of data that has been collected” (Abma et al., 2019, p. 158). Concretely, it was only through the joint data interpretation that several limitations were uncovered. The quantitative results alone were therefore only used with caution to answer the question of the influence of the participatory approach on the three selected implementation outcomes.

### Conclusion

For more than 40 years, the importance of stakeholder participation in health contexts has been recognized and formally declared in the World Health Organization’s (WHO) Alma Ata Declaration (Hendricks et al., 2018). Stakeholder participation is “increasingly considered best research practice to improve care management services” (Kowe et al., 2020, p. 908). While there are some examples of stakeholder participation in dementia care research (Coffey et al., 2021; Dupuis et al., 2021; Goh, 2021; Tan et al., 2021; Watchman et al., 2021), it has not been adopted on a larger scale yet (Kowe et al., 2020). With our study, we contributed to alleviate this lack of studies by incorporating a participatory approach in dementia care research. By including stakeholders in various steps of the study, the research is more in accordance with ethical standards as it is more synchronized with the needs of the care population (Forsythe et al., 2019; Hemphill et al., 2020; Staley & Minogue, 2006). Also, in their role as local healthcare experts from different healthcare sectors in the region, the stakeholders will continue to work with PwD and caregivers thus making the research findings “better aligned with real-world clinical needs” (Hemphill et al., 2020, p. 328) and more sustainable. The lessons learned from this study will influence the set-up of the subsequent RoutineDeCM study, so resources will be appointed to relevant, high priority topics (Forsythe et al., 2019; Kowe et al., 2020; Staley & Minogue, 2006) which can lead to faster changes in healthcare delivery (Forsythe et al., 2019).

Future studies should focus on the inner psychological aspects of stakeholders during a research process. Possible cognitive biases or their role perception can influence stakeholders’ assessment of the implementation outcomes and their view on any kind of healthcare implementation significantly.
